# Supplementary material for: Ubiquitylation of MLKL at lysine 219 positively regulates necroptosis-induced tissue injury and pathogen clearance
Source: Nat Commun. 2021 Jun 7;12:3364. doi: 10.1038/s41467-021-23474-5 (PMC8184782; doi:10.1038/s41467-021-23474-5)
Supplement: Supplementary file 1 — Supplementary Information [file 41467_2021_23474_MOESM1_ESM.pdf]

Supplementary Figures

Ramos Garcia et al., 2021

Ubiquitylation of MLKL at lysine 219 positively regulates necroptosis-induced tissue injury and pathogen clearance

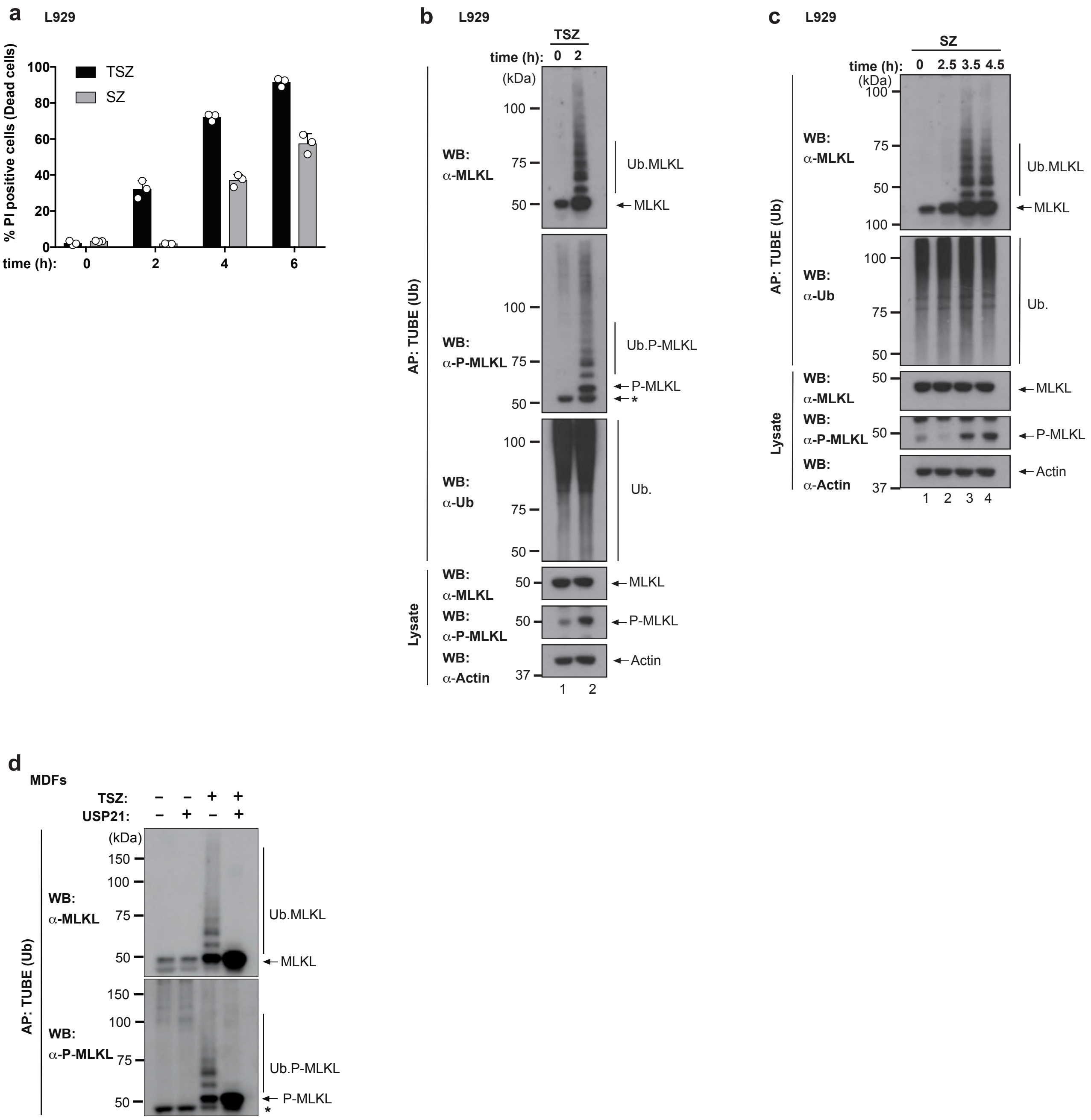

Supplementary Fig. 1
Ramos Garcia et al, 2021

Supplementary Fig.1. Endogenous MLKL is ubiquitylated during necroptosis.

a. Quantification of propidium iodide positive (PI^+) L929 cells following treatment with SM-164 (100 nM) and z-VAD-FMK (20 μM) in presence or absence of TNF (10 ng/ml), (TSZ/SZ) for the indicated times. Results are representative of those from two independent experiments with three technical repeats. Data is presented as mean±SD.

b. Tandem Ubiquitin Binding Entities (TUBE) affinity purification (AP) of ubiquitylated proteins from L929 cells following treatment with TSZ. * refers to non-specific bands.

c. L929 cells were treated with SZ for the indicated time points. Lysates were subjected to TUBE AP and the presence of ubiquitylated MLKL was determined by immunoblot analysis.

d. TUBE AP of MDFs treated with TSZ or left untreated. Prior to elution from the beads, samples were split in two and incubated with or without 2 μM of USP21. * refers to non-specific bands. Source data are provided as a Source Data File.

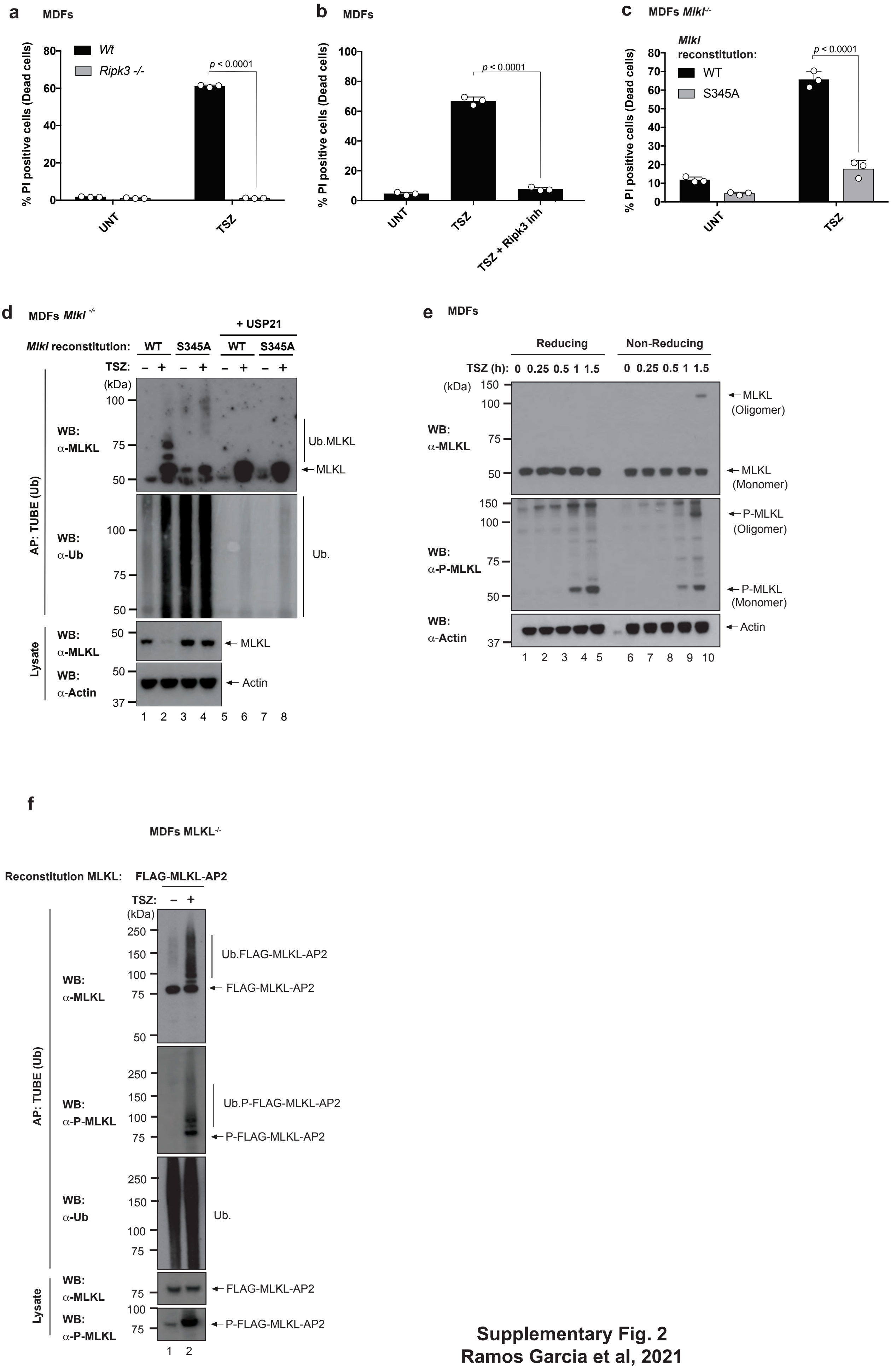

Supplementary Fig. 2
Ramos Garcia et al, 2021

Supplementary Fig.2. Role of RIPK3-mediated phosphorylation In MLKL ubiquitylation.

- **a.** Quantification of propidium iodide positive (PI^{+}) *Ripk3*$^{-/-}$ and WT MDFs treated with TNF (10 ng/ml), SM-164 (100 nM) and z-VAD-FMK (20 μM) (TSZ) for 2.5h.
- **b.** Quantification of PI^{+} MDFs treated with TSZ (as in a) in presence or absence of RIPK3 inhibitor (RIPK3i GSK'843 2 μM).
- **c.** Quantification of PI^{+} *Mlkl*$^{-/-}$ and WT MDFs reconstituted with *Mlkl*^{S345A} or *Mlkl*^{WT}. Expression of MLKL transgene was induced with doxycycline (DOX) and subsequently cells were treated with TSZ. In Supplementary Figure 2a-c, n= 3 wells/group. The results are representative of those from one independent experiment with three technical replicates each. Data is presented as mean±SD. Statistical analysis shown was calculated by two-way ANOVA with Sidak's multiple comparison test in Supplementary Figure.2a and 2c and one-way ANOVA in Supplementary Figure. 2b.
- **d.** Tandem Ubiquitin Binding Entities (TUBE) affinity purification (AP) of ubiquitylated proteins from *Mlkl*$^{-/-}$ MDFs reconstituted with *Mlkl*^{WT} or *Mlkl*^{S345A}, and stimulated with TSZ after DOX pretreatment. Samples were split in two and incubated with USP21.
- **e.** Oligomerisation assessment of MLKL in MDFs treated with TSZ. The samples were split in two and either eluted with a sample buffer containing β-mercaptoethanol (reducing condition) or without it (non-reducing condition) to enable visualization of MLKL trimers.
- **f.** TUBE AP of ubiquitylated proteins from *Mlkl*$^{-/-}$ MDFs reconstituted with N-terminally FLAG-tagged MLKL. The construct is also tagged with APEX2 (AP2) biotin ligase on the C-terminal. The results in Supplementary Figures 2d-f are representative of those from two (2d-e) or one (f) independent experiment. Source data are provided as a Source Data File.

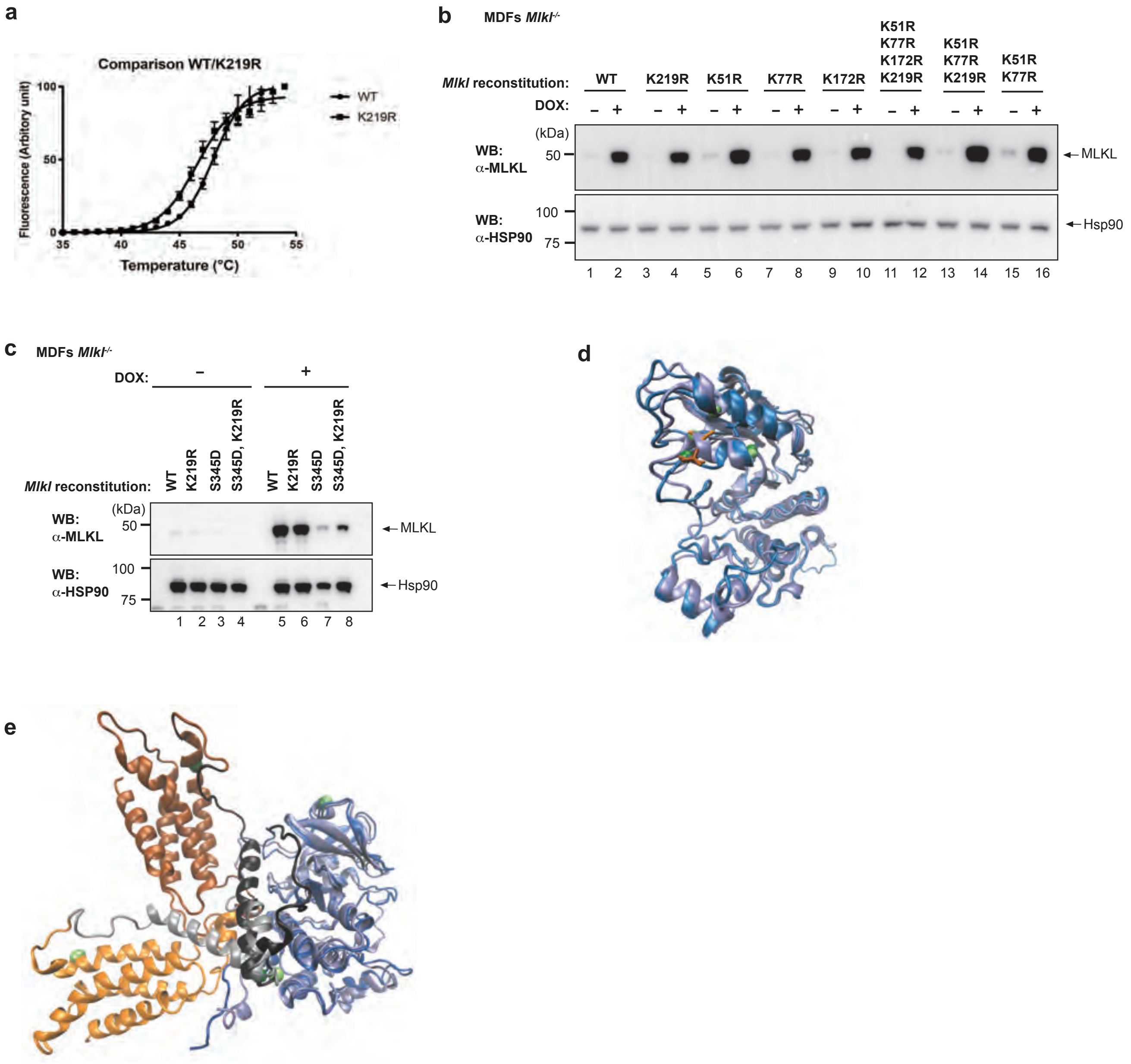

Supplementary Fig. 3
Ramos Garcia et al, 2021

Supplementary Fig.3. MLKL mutant constructs characterization

- **a.** Thermal shift assay of $MLKL^{WT}$ and $MLKL^{K219R}$. Data are presented as mean values ±SD and are representative of two independent experiments.
- **b.** *Mlkl*$^{-/-}$ MDFs were reconstituted with the indicated *Mlkl* K mutants and their expression was induced by treatment with doxycycline (DOX, 0,1 µg/ml for 3 h). MLKL expression was analysed by western blot.
- **c.** Evaluation of MLKL expression in reconstituted *Mlkl*$^{-/-}$ MDFs. *Mlkl*$^{-/-}$ MDFs were reconstituted with the respective *Mlkl* mutants and their expression was induced by treatment with DOX (0.1 µg/ml for 5 h). MLKL expression was analysed by western blot. Supplementary data 3b-c are representative of one independent experiment.
- **d.** MLKL activation loop helix in crystal structure and S345phos simulations. The pseudokinase domains from the crystal structure (light blue) and a snapshot from the S345phos simulations (dark blue) are aligned. S345, shown in orange is pointed toward the adjacent helix and T235 in the crystal structure (light orange) and is pointed out toward the solvent in the S345phos simulations (dark orange). The activation loop helix unwinds and changes orientation with respect to the adjacent helix in the simulations with S345 phosphorylated. Green spheres show the residues (232, 339, 346) that are used to measure the angle between the activation loop helix and adjacent helix (35° in the crystal structure and 52° in the S345phos snapshot shown).
- **e.** Range of conformations that the 4HB domain samples in WT MD simulations. Two snapshots from the WT simulations are aligned at the pseudokinase domain. The more open conformation (4HB domain angle = 101°) is shown in light blue (pseudokinase domain), light grey (brace helices), and light orange (4HB domain), while the more closed conformation (4HB domain angle = 39°) is shown in dark blue, black, and dark orange. Green spheres show the residues (113, 155, 189) that are used to measure the 4HB domain angle. Source data are provided as a Source Data File.

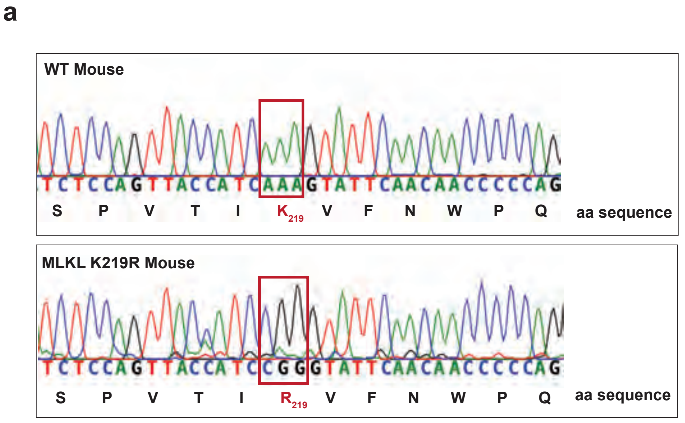

b

Total nºmice: 69

Mlkl Genotype	Expected nº(%)	Observed nº(%)
WT/WT	17.25 (25)	14 (20.3)
K219R/K219R	17.25 (25)	19 (27.5)
WT/ K219R	34.5 (50)	36 (52.2)

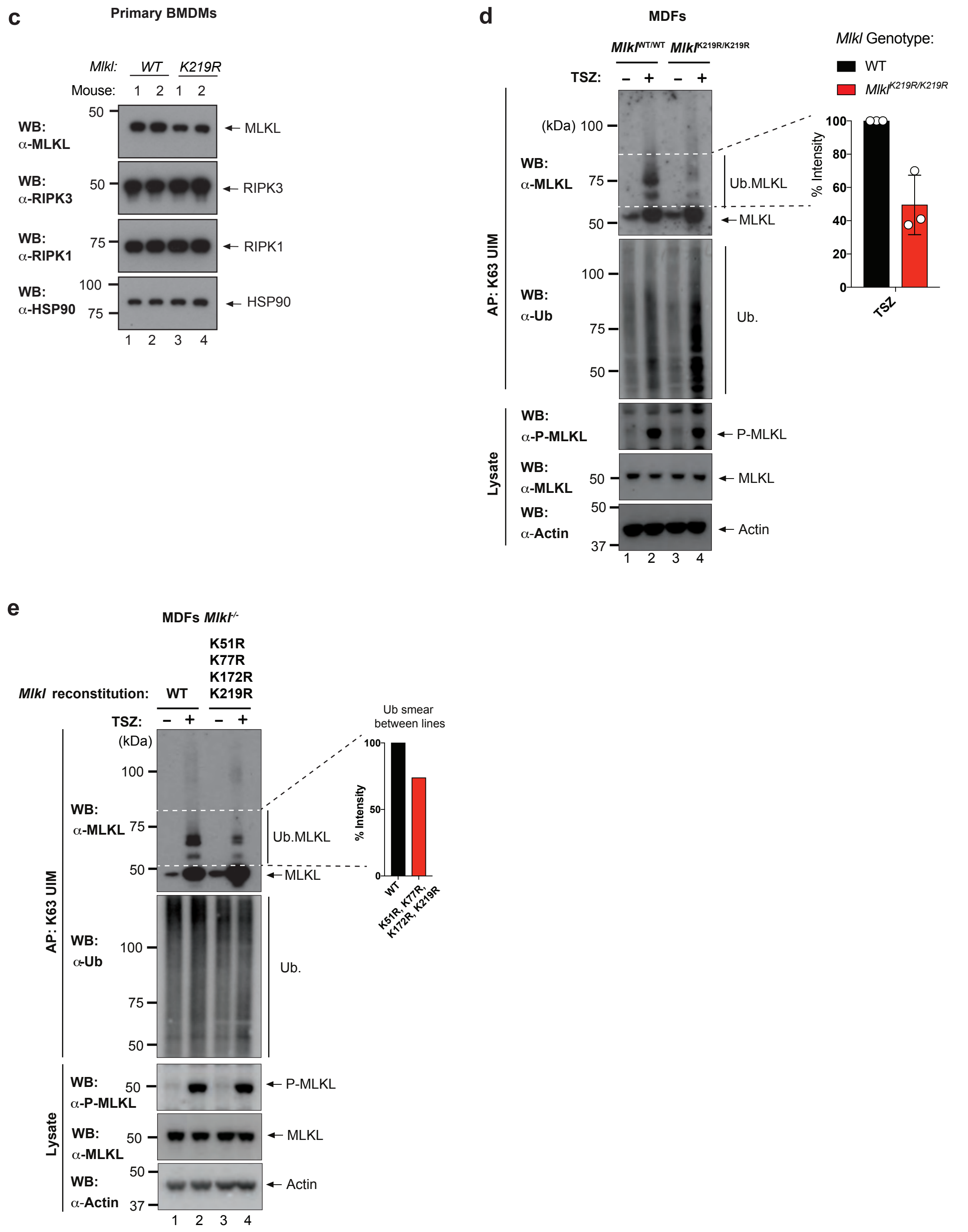

Supplementary Fig. 4
Ramos Garcia et al, 2021

Supplementary Fig.4. Characterization of cells derived from *Mlkl*$^{K219R/K219R}$ *mice.*

a. Validation of the of the *MlklK*$^{219R/K219R}$ knock-in allele. To mutate the amino acid (aa) at position 219 from K to R, we introduced the AAA to CGG mutation.

b. Expected and observed Mendelian inheritance after breeding *Mlkl*$^{WT/K219R}$ X *Mlkl*$^{WT/K219R}$ animals. New-born animals were genotyped, and the observed and expected genotypes were annotated, confirming that the *Mlkl*$^{K219R/K219R}$ mice were born at expected Mendelian inheritance. Data were analysed in GraphPad Prism 7 with statistical test by chi-square goodness-of-fit test.

c. Western blot analysis with the indicated antibodies of primary BMDMs derived from mice with the indicated genotypes. Data is representative of one independent experiment with two biological repeats (2 mice).

d. K63-UIM affinity purification (AP) of ubiquitylated proteins from *Mlkl*$^{WT/WT}$ and *Mlkl*$^{K219R/K219R}$ MDFs following treatment with TNF (10 ng/ml), SM-164 (100 nM) and z-VAD-FMK (20 μM) (TSZ) for 2.5h. The graph shows the quantification of the smear in the three independent experiments that were conducted. Data are presented as mean values ±SD.

e. K63-UIM AP of ubiquitylated proteins from *Mlkl*$^{-/-}$ MDFs reconstituted with the indicated mutants. Cells were pre-treated with doxycycline following treatment with TSZ for 2.5h. Data is representative of one independent experiment. Source data are provided as a Source Data File.

a

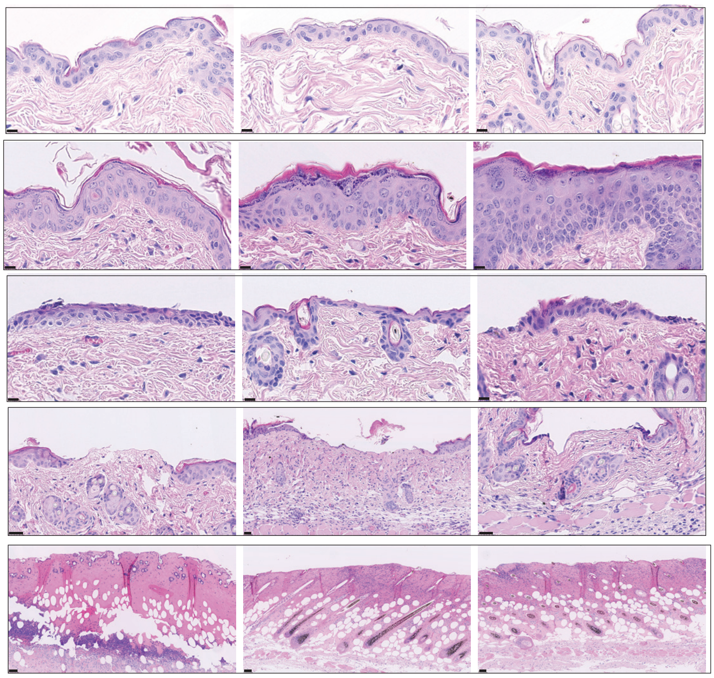

Score 0: Normal epidermis with no changes to: Stratum corneum, Stratum lucidum, Stratum granulosum, Stratum spinosum, Stratum basale

Size of affected region: 0-100%

Score 1: Thickening of epidermis with (miled to marked) degree of changes to any of the strata: Stratum corneum, Stratum lucidum, Stratum granulosum, Stratum spinosum, Stratum basale. Stratum corneum can be lifted away from the underlain epidermis.

Size of affected region: 0-100%

Score 2: Epidermal erosion, partial loss of the epidermis with the stratum basale left intact.

Size of affected region: 0-100%

Score 3: Ulcer-loss of epidermis, including the stratum basale.

Size of affected region: 0-100%

Score 4: Ulceration of the epidermis. The underlying dermis shows formation of dense collagenous fibrous tissue, which is more prominent in dermis but appears extending to the hypodermis.

Size of affected region: 0-100%

Histopathological (multivariate) Lesion Score (HLS)
[(0x % Score 0) + (1x % Score 1) + (2x % Score 2) + (3x % Score 3) + (4x % Score 4)]
min=0; max=400

b

FACS Spleen analysis

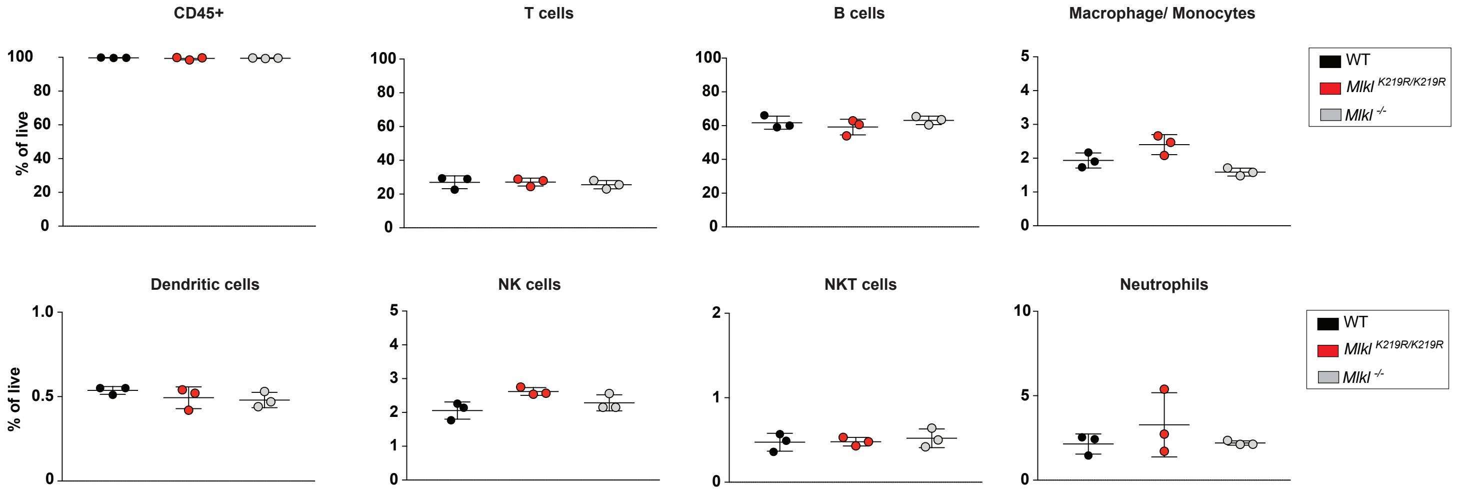

Supplementary Fig. 5
Ramos Garcia et al, 2021

Supplementary Fig.5. Method for the assessment of the skin lesions and immune landscape characterization in *Mlkl*$^{K219R/K219R}$ mice.

- **a.** Characterization of the Histopathological (multivariate) Lesion Scoring (HLS) system: Assessment of lesions in the epidermal and immediate dermal level, according to increasing severity with representative H&E images. Scale bars for score 0-2, 10 μM; 25 μM for score 3 and 50 μM for score 4.
- **b.** FACS analysis characterizing the immune infiltrate in steady-state in the spleen of *Mlkl*$^{WT/WT}$, *Mlkl*$^{K219R/K219R}$ *and Mlkl*$^{-/-}$ mice. Data shows the results derived from n=3 mice per genotype from one independent experiment. Data are presented as mean values ±SD. Source data are provided as a Source Data File.

a ASTX660 + Emricasan

Mlkl$^{WT/WT}$

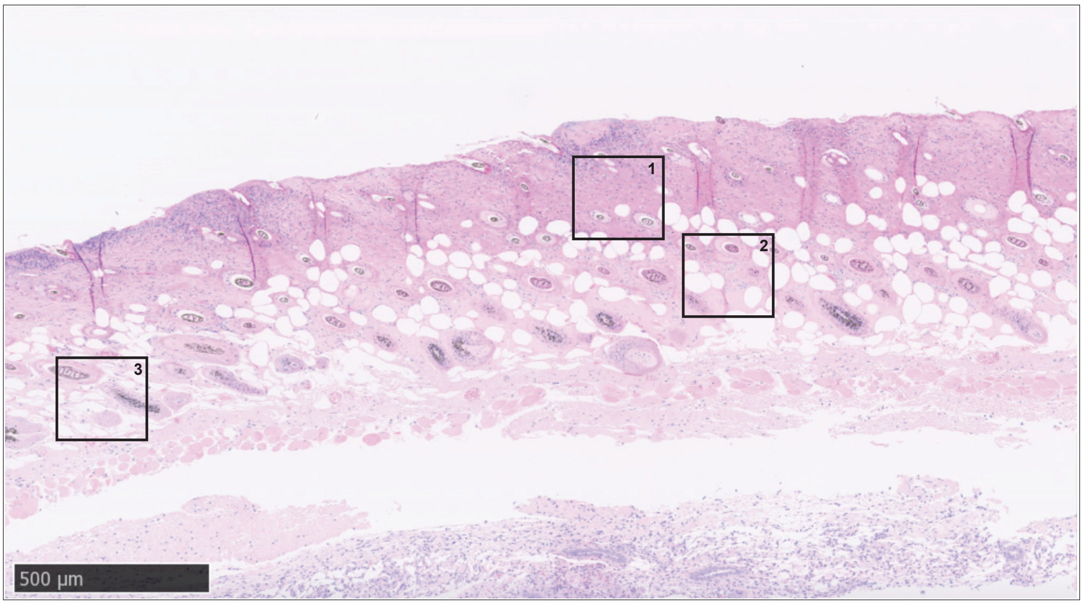

Magnification:

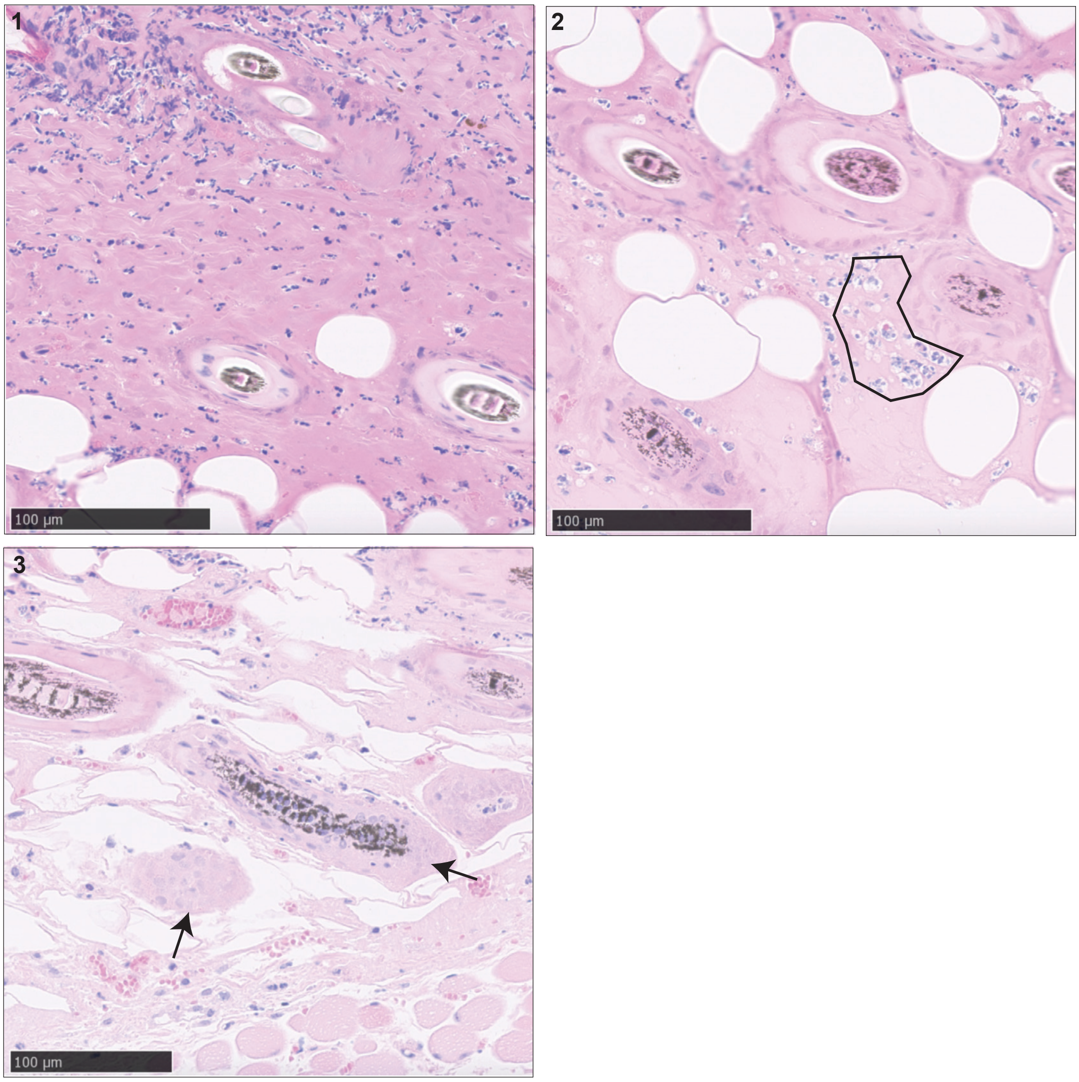

Supplementary Fig. 6
Ramos Garcia et al, 2021

Supplementary Fig.6. Representative H&E images of skin lesion in WT mice following injection with ASTX660/ Emricasan (AE)

a. Representative H&E images of *Mlkl*$^{WT/WT}$mice treated with AE. Magnifications showing cellular necrosis. Data is representative of lesions observed in n=9 mice, tested in two independent experiments.

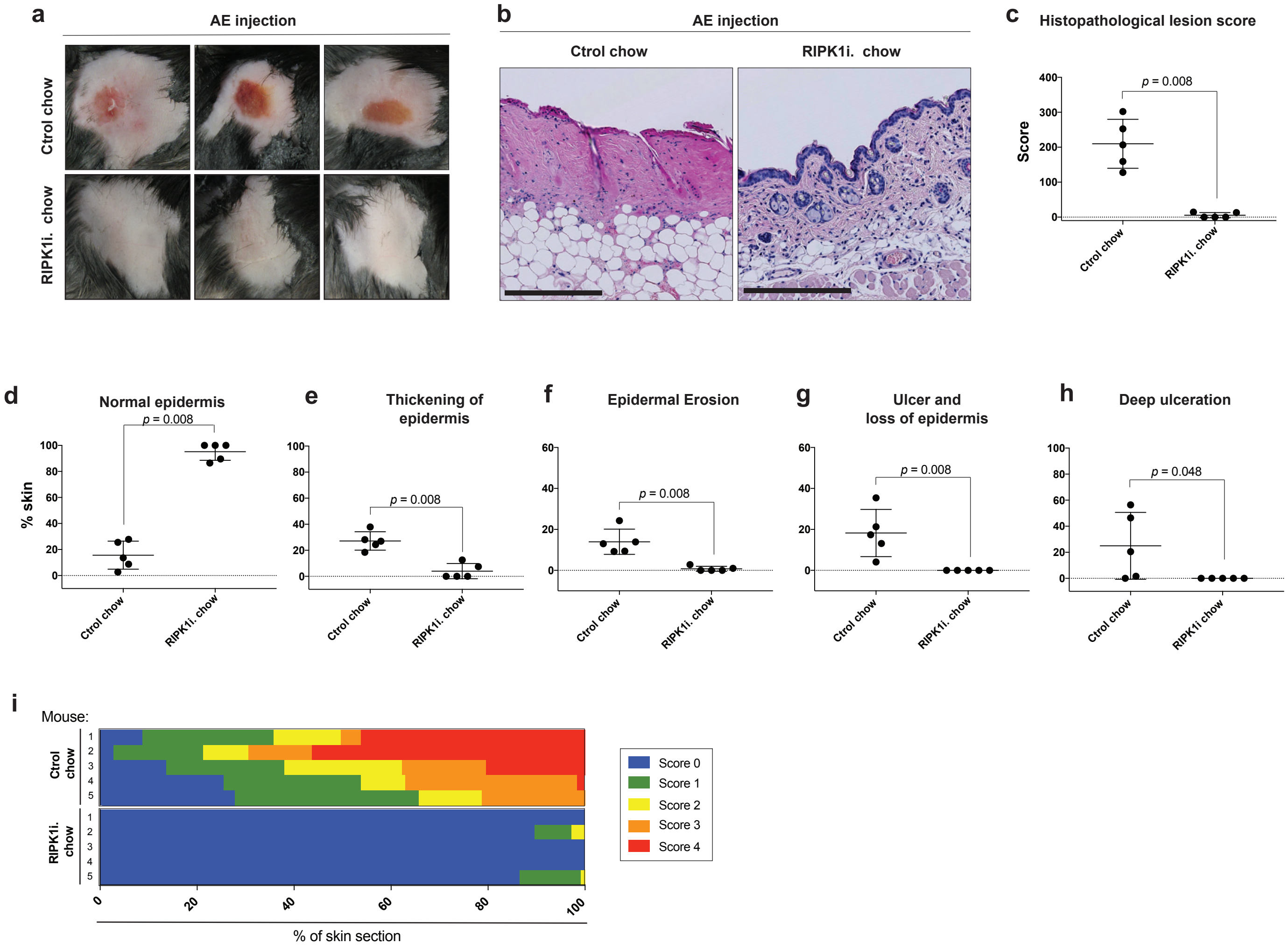

Supplementary Fig. 7
Ramos Garcia et al, 2021

Supplementary Fig.7. AE-induced skin injury is dependent on RIPK1 kinase activity.

a. Representative macro images of skin lesions from *Mlkl*$^{WT/WT}$ mice 72 hrs after injection with ASTX660/ Emricasan (AE) fed chow with RIPK1i (n = 5) or control chow (n = 5). Chow feeding with RIPK1i-chow or control chow started seven days prior to AE injection.

b. Representative images of skin sections of mice treated as in a. H&E staining of lesions occurring 72 h post-injection. Scale bars, 250 μM.

c. A final histopathological multivariate Lesion Score (HLS) of mice treated as in a. The calculation considered the proportional score (%) of given skin lesions within each sample. This was then multiplied by a power score reflecting lesion severity.

d. Percentage contribution of regular epidermis with no changes to Stratum corneum, Stratum lucidum, Stratum granulosum, Stratum spinosum, and Stratum basale.

e. Percentage contribution of epidermis thickening with (mild to marked) degree of changes to any of the strata: Stratum corneum, Stratum lucidum, Stratum granulosum, Stratum spinosum, Stratum basale.

f. Percentage contribution of epidermal erosion or partial loss of the epidermis, with the Stratum basale left intact.

g. Percentage contribution of ulceration.

h. Percentage contribution of ulceration with dermal and hypodermal fibrosis and evidence of cell death. Extensive necrotic area and the presence of keratotic debris. In graphs d-h the amount of effected region was scored from 0-100 %. In graphs c-h n=5 mice per group and the score for each mouse is represented by a single dot. Data is presented as mean±SD. The results show data collected in one independent experiment. Statistical significance by two-sided non-parametric Mann-Whitney test.

i. Percentage of individual skin characteristics of mice treated as in a. Assessment was performed on the entire length of the skin sample for each genotype. Two skin sections from each mouse were assessed. Source data are provided as a Source Data File.

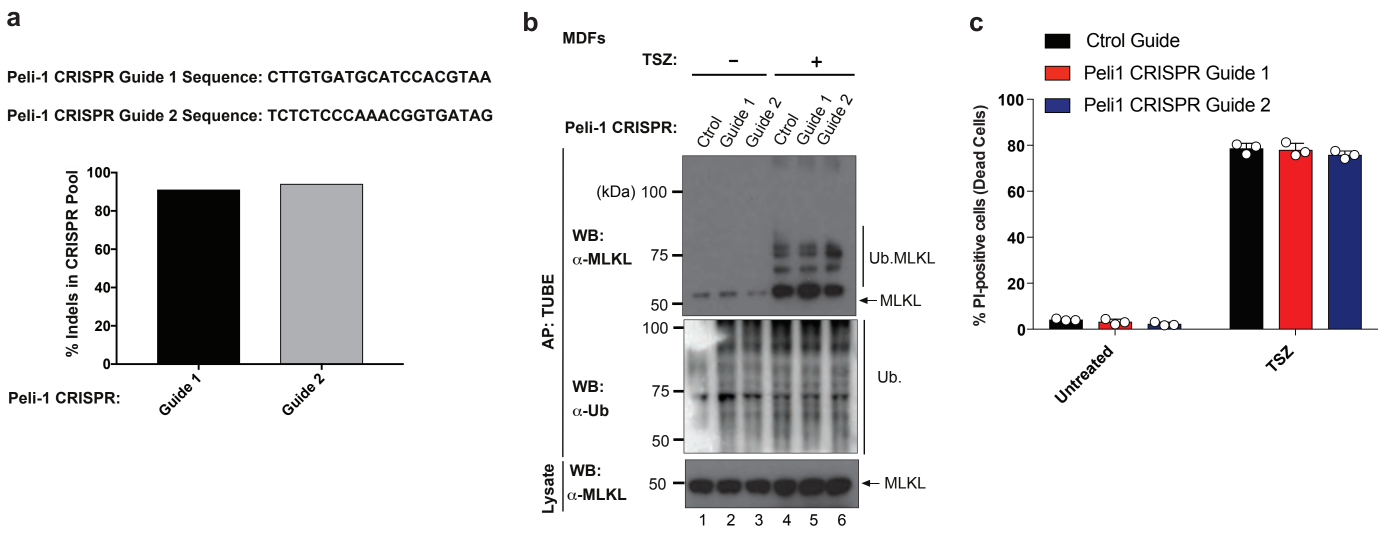

Supplementary Fig. 8
Ramos Garcia et al, 2021

Supplementary Fig.8. Peli-1 deletion in MDFs does not affect MLKL ubiquitylation.

- **a.** % of indels in two MDFs CRISPR pools targeting Peli-1. Both of the CRISPR guides resulted in high indel efficiency (91 % and 94 % for *guide 1* and *guide 2*, respectively). Analysis was conducted via https://ice.synthego.com.
- **b.** Tandem Ubiquitin Binding Entities (TUBE) affinity purification (AP) of ubiquitylated proteins from control pool and Peli-1 CRISPR pools following treatment with TNF (10 ng/ml), SM-164 (100 nM) and z-VAD-FMK (20 μM) (TSZ) for 2h. Data shown is representative of one independent experiment.
- **c.** Quantification of propidium iodide positive (PI^+) MDFs treated with TSZ for 2.5 h. n=3 wells/group. Data is representative of those from two independent experiments with three technical repeats each. Data is presented as mean±SD. Source data are provided as a Source Data File.

Supplementary Table 1. List of primers used in this study

Mutagenesis:	Primer Sequence (5' > 3')
mMLKL S345A	F: TTAAGCAAAACACAGAATGCCATCAGCCGGACAGCAAAG
	R: CTTTGCTGTCCGGCTGATGGCATTCTGTGTTTTGCTTAA
mMLKL S345D	F: TTAAGCAAAACACAGAATGACATCAGCCGGACAGCAAAG
	R: CTTTGCTGTCCGGCTGATGTCATTCTGTGTTTTGCTTAA
mMLKL K219R	F: AGATCTCCAGTTACCATCAGAGTATTCAACAACCCCCAG
	R: CTTTGCTGTCCGGCTGATGTCATTCTGTGTTTTGCTTAA
mMLKL K219M	F: AGATCTCCAGTTACCATCATGGTATTCAACAACCCCCAG
	R: CTGGGGGTTGTTGAATACCATGATGGTAACTGGAGATCT
mMLKLK 172R	F: CTGAAGCAATGCTCACTAAGACCCACACAGGAGATCCCA
	R: TGGGATCTCCTGTGTGGGTCTTAGTGAGCATTGCTTCAG
mMLKL K51R	F: CTCCAGGCCCAAGGAAAGAGGAACCTGCCCGATGACATTACT
	R: AGTAATGTCATCGGGCAGGTTCCTCTTTCCTTGGGCCTGGAG
mMLKL K77R	F: GCTAACCAGCAGATAGAAAGATTCAGCAAG
	R: CTTGCTGAATCTTTCTATCTGCTGGTTAGC

Seq. primers	Primer Sequence (5' > 3')
mMLKLseq _for	ATGGATAAATTGGGACAGATCATCAAGTTA
mMLKLseq_rev	TTACACCTTCTTGTCCGTGGATTCTTCAAC
pBSEQ_for	TTGTGAGCGGATAACAATTTCACACAGGAA
pBSEQ_rev	AAACGACGGCCAGTGAGCGCGCGTAATAC
TRIPZ5'SQ	TTCAGTACTTTACAGAATCGTTGCCTG
TIPZSQ_F	GAGAACGTATGTCGAGGTAGGCGTGT
